# Computed Tomography of the Hyoid Apparatus in Equine Headshaking Syndrome

**DOI:** 10.3390/vetsci12060511

**Published:** 2025-05-23

**Authors:** Ralph A. Lloyd-Edwards, Eva Mulders, Marianne M. Sloet van Oldruitenborgh-Oosterbaan, Stefanie Veraa

**Affiliations:** 1 Diagnostic Imaging, Department of Clinical Sciences, Faculty of Veterinary Medicine, Utrecht University, Yalelaan 1, 3584 CL Utrecht, The Netherlands; 2 VET.CT, Broers Building, 21 JJ Thomson Avenue, Cambridge CB3 0FA, UK; 3 Department of Clinical Sciences, Faculty of Veterinary Medicine, Utrecht University, Yalelaan 1, 3584 CL Utrecht, The Netherlands; 4 Internal Medicine, Department of Clinical Sciences, Faculty of Veterinary Medicine, Utrecht University, Yalelaan 1, 3584 CL Utrecht, The Netherlands; 5 Eikenlust Equine Consultancy, Kees Boekelaan 8, 3723 BA Bilthoven, The Netherlands

**Keywords:** CT, horse, trigeminal-mediated headshaking, diagnostic imaging, veterinary

## Abstract

**Introduction:** Headshaking is a common condition in horses, most cases are presumed idiopathic/trigeminal-nerve mediated. Diagnostic work-up of a headshaking horse may involve computed tomography (CT) of the head to exclude causative structural pathology. The relevance of the presence and severity of hyoid apparatus findings at CT to headshaking is unknown. **Materials and methods:** A retrospective analysis of CT changes in the hyoid apparatus in horses was carried out. Comparisons were performed between horses with signs of headshaking and a control population and a subgroup of horses with signs of headshaking and no other ‘likely relevant findings’ to headshaking and the control population. **Results:** The grade of temporohyoid joint sheath ossification, mineralisation of the tympanohyoid cartilage, and widening and narrowing of the temporohyoid joint all showed significant correlation with age. Findings of the remaining hyoid apparatus (fracture, deformation, or arthropathy) showed significant correlation with temporohyoid joint grade. Centres of ossification of the epihyoid, thyrohyoid, and lingual processes were described. No consistent association of headshaking to hyoid changes was found. Odds ratios were increased in many cases, particularly in comparisons of the subgroup with no ‘likely relevant findings’; however, statistical significance was not reached. **Conclusions:** CT findings of the temporohyoid joint are not consistently associated with clinical signs of headshaking.

## Simple Summary

Headshaking is a common and debilitating condition in horses. Most cases have no structural cause identified, so are presumed functional hypersensitivity of the trigeminal-nerve. Diagnostic work-up of a headshaking horse may involve computed tomography of the head to exclude any causative structural abnormalities. The hyoid apparatus is a series of bones that support the tongue and larynx and are connected with the skull via the temporohyoid joint. Abnormalities of the hyoid apparatus are reported to cause headshaking; however, the relevance of changes at computed tomography are not completely understood. The study retrospectively examined many computed tomographic examinations of horses with and without headshaking. Many changes in the hyoid apparatus were characterised. Significant associations between changes of the temporohyoid joint, remaining hyoid apparatus, and age were found. No changes of the temporohyoid joint at computed tomography significantly increased the risk of headshaking. The information gained from the study can be applied to clinical interpretation of the computed tomography of the hyoid apparatus in instances of headshaking.

## 1. Introduction

Headshaking is a common clinical presentation in horses with potentially serious implications to welfare, safety, and exercise [1,2]. Headshaking is characterised by spontaneous and uncontrollable episodes of repetitive movements of the head and neck; however, it shows a wide range of variation of signs regarding the type of head movement, timing of the movements, and additional features such as snorting, rubbing, or striking [1,3]. Horses exhibiting signs of headshaking fall broadly into two categories: those with a pathology presumed to be causative for the clinical signs and, more often, those with no identified causative pathology [3,4]. Horses with signs of headshaking and no identified causative pathology are termed ‘idiopathic’ or trigeminal-nerve mediated, and they are likely functional in aetiology with a lowered activation threshold for the infraorbital branch of the trigeminal nerve [5,6]. Diagnosis of trigeminal nerve-mediated headshaking is predominantly a diagnosis of exclusion. Computed tomography (CT) can provide additional information to help identify or exclude structural pathologies of the head which may be associated with headshaking signs [4,5,7]. However, the utility of CT is under discussion, partly due to the difficulty in deciding the relevance of a finding to the clinical presentation, with limited comparison studies available [4,5,7,8,9,10].

The hyoid apparatus is a system of paired and unpaired bones which support the tongue, pharynx, and larynx and are connected with the skull via the temporohyoid joint [11,12]. Changes in the temporohyoid joint in particular are reported to cause clinical signs including headshaking [13,14,15,16]. A broad range of joint changes are reported at CT [12,17]. The temporohyoid joint is cartilaginous involving the stylohyoid bone, the tympanohyoid cartilage, and the temporal bone. The tympanohyoid cartilage attaches the stylohyoid bone to the styloid process of the temporal bone and is surrounded by the styloid process sheath [12]. Variable mineralisation of the styloid process sheath and the tympanohyoid cartilage, changes in the size of the joint, and associated fractures or otitis media are reported with variable relevance [11,12,17,18,19,20]. The degree of new bone formation can be graded from 0 to 3 [17,21], and grade is associated with age [12]. Multiple variations of the remaining hyoid apparatus, including fusion of the epihyoid and stylohyoid as well as ossification centres of the basihyoid, are also reported, presumed to be anatomical variations without clinical significance [11].

Multiple studies have examined the relationship between changes in the temporohyoid joint and clinical signs of temporohyoid osteopathy [16,17,19,22]. However, they do not have a specific focus on headshaking and were published before recent insights into the anatomy and morphology of the hyoid apparatus [11,12]. The increased clarity and understanding of the anatomy and presumed anatomical variations allow further assessment of changes in the hyoid apparatus. Specifically, the differentiation of changes in the tympanohyoid cartilage and surrounding styloid process sheath could aid in assessment of the joint.

In the current study, we aim to specifically evaluate structural CT changes in the hyoid apparatus with the presence of headshaking. This could give clinicians additional valuable information in the characterisation, distribution, and relevance of different imaging findings of the hyoid apparatus with respect to headshaking.

The null hypothesis proposes that changes in the hyoid apparatus identified on CT do not show a statistically significant association with headshaking.

## 2. Materials and Methods

All horses referred to the Diagnostic Imaging Division, Department of Clinical Sciences of the Veterinary Teaching Hospital, Faculty of Veterinary Medicine of Utrecht University with reported clinical signs consistent with headshaking between February 2015 and June 2022 were examined. The selection was based on the appearance of keywords or phrases within the clinical history provided for imaging in the PACS (Enterprise Imaging, AGFA, Septestraat 27, 2640 Mortsel, Belgium). The cases were then confirmed to display signs of headshaking by evaluation of the clinical records by a bachelor-degree veterinary student (E.M.) under supervision of a diplomate of the European College of Equine Internal Medicine (M.S). In instances of multiple examinations of the patient, the first examination within the selection period when signs of headshaking were present was utilised.

The CT studies were assessed by a diplomate of the European College of Diagnostic Imaging (R.A.L.-E.). Studies were excluded if a complete examination of the hyoid examination was not possible; however, an optimal examination of head/cranial neck was not required (i.e., movement artifacts or partially limited field of view outside of the hyoid apparatus). Horses younger than one year of age were also excluded.

Horses were first assessed for the presence of a finding considered likely relevant to headshaking in the CT study, excluding the hyoid apparatus. A classification system of likely relevant findings with respect to headshaking was derived from the classification presented by Perrier et al. [5]. The definitions of the classification system were broadened with consideration of recent relevant literature. For example, mineralisation of the odontoid ligament and cholesterinic granulomas were not included as likely relevant findings with respect to headshaking due to recent publications [8,10]. Changes in the infraorbital canal were not included as a likely cause as the only changes with reported potential importance occur in horses with sinusitis which were already excluded regardless of infraorbital canal changes [9,23].

The following abnormalities were considered likely relevant to headshaking, and horses with these detected were not included in the subgroup of headshaking horses with ‘no likely relevant findings’.

- Dental abnormality—any excluding infundibular gas [24,25].
- Sinus abnormality—sinusitis/mass lesions (cyst/ethmoidal haematoma/neoplasia) or osseous modelling/deformation [26].
- Skull lesion—mass, osteomyelitis, fracture (excluding the hyoid or adjacent temporal bone), or severe temporomandibular joint disease.
- Intracranial lesion (excluding cholesterinic granulomas [10]) or an abnormality of a cranial nerve.
- Middle/inner ear abnormality.
- Nuchal bursitis.

Detailed examination of the hyoid region of all horses was then performed. Recorded features included:

- Temporohyoid grade: The grade of new bone formation within the stylohyoid sheath was graded using the previously reported 0–3 grading system [17,21]. In our study, grade two or three required new bone formation crossing both sides of the joint, as this was not clearly defined in the original scheme (see below).
  o Grade 0—absent or a minimal/equivocal amount of new bone formation on either side of the joint.
  o Grade 1—new bone formation on either side not crossing the joint.
  o Grade 2—new bone formation crossing both sides of the joint and extending less than 10 mm.
  o Grade 3—new bone formation crossing both sides of the joint and extending over 10 mm.
- Other features of the temporohyoid joint:
  o Mineralisation—the presence of any mineral attenuation within the region of the tympanohyoid cartilage.
  o Gas—any gas attenuation within the region of the tympanohyoid joint.
  o Narrowing—subjective decrease in height of the temporohyoid joint space/cartilage.
  o Widening—subjective increase in width of the temporohyoid joint.
  o Fusion—complete osseous fusion of the temporal and stylohyoid bones.
- Remaining hyoid apparatus: Fracture of the hyoid bones or temporal bone or luxation of the hyoid joints. Arthropathy of the hyoid joints. Osseous deformation or fusion of hyoid joints. Presence of a separate ossified epihyoid. Multiple centres of ossification of the lingual process. Fusion of the thyrohyoid and basihyoid bones.

A control group of horses was selected from horses presenting for a CT examination of the head to the Division of Diagnostic Imaging for conditions other than headshaking between October 2018 and December 2020. Similar exclusion criteria with respect to study quality and horse age were used. In this group, only the hyoid assessment was performed. Changes outside of this region were not assessed as exclusion of other causes of headshaking was not required.

Statistical analysis was performed using SPSS (IBM Corp. Released 2023. IBM Statistics for Windows Version 29.0 Armonk, NY, USA, IBM Corp). Descriptive statistics and objective analysis were performed to assess the data, including frequency of the changes in the hyoid in both headshaking and control groups [17]. Analysis was performed before and after exclusion of horses with another ‘likely relevant finding’ within the headshaking group. Two-sided Fisher’s exact tests were used to compare nominal data, as well as the independent-sample Mann–Whitney U test for comparisons of independent ordinal data divided by a nominal variable and Spearman’s rank correlation coefficient for comparison of related ordinal data. Odds ratios were also calculated for appropriate nominal variables, stating the relative odds of headshaking between horses with a finding present or absent.

## 3. Results

### 3.1. Signalment of Group

A total of 141 horses met the inclusion criteria within the headshaking group and 149 in the control group. The median age was 9 years (range 3–23) in the headshaking group and 10 (range 1–24) in the control group. The number of mares, geldings, and stallions was similar in headshaking (*n* = 55, *n* = 86 and *n* = 0) and control (*n* = 65, *n* = 73 and *n* = 11) groups, respectively. The majority of included horses were Dutch warmbloods or riding horses within both headshaking (*n* = 80) and control (*n* = 72) groups. The remaining sample was heterogeneous with many other breeds.

### 3.2. Temporohyoid Findings and Correlation with Signalment

The maximum temporohyoid grade was defined as the highest grade observed on either the right or left side (Table 1). The distribution of grades was similar on the right (41.0% Grade 0, 47.9% Grade 1, 4.1% Grade 2, and 6.9% Grade 3) and left sides (39.7% Grade 0, 49.7% Grade 1, 4.8% Grade 2, and 5.9% Grade 3). Most horses had symmetrical temporohyoid grades, with less than 25% having a different grade on right and left sides (Table 1).

The temporohyoid grade significantly increased with age (Spearman’s rank coefficient 0.2, *p* < 0.001). Horses with temporohyoid Grade 0 had a median age of 8 (range 1–23), the value for Grade 1 was 9 (range 1–23), that for Grade 2 was 9 (range 4–19), and that for Grade 3 was 13 (range 6–24). The difference in temporohyoid grade between left and right sides showed no correlation with age (Spearman’s rank coefficient 0.077, *p* = 0.19).

The maximum temporohyoid grade did not vary between mares and stallions/geldings (independent sample Mann–Whitney U test *p*-value of 0.642 and *r*-value −0.027).

Mineralisation of the tympanohyoid cartilage was detected frequently (Table 1) and when present was usually bilateral (85 of 135 horses). The presence of mineralisation (unilaterally or bilaterally) significantly increased with age (independent sample Mann–Whitney U test, *p* = 0.001, *r* = 0.23), with a median age of 11 years in horses with unilateral or bilateral mineralisation and 9 years without mineralisation present.

Temporohyoid grade varied significantly with mineralisation of the tympanohyoid cartilage (independent sample Mann–Whitney U test, *p* < 0.001, *r* = 0.27), as 35 of 94 (37%) of horses with a maximum temporohyoid grade of 0 had tympanohyoid mineralisation compared to 26 of 26 (100%) with Grade 3.

Gas within the temporohyoid joint was detected infrequently; it had significant association (independent sample Mann–Whitney U tests) with temporohyoid grade (*p* < 0.001, *r* = 0.23) but not age (*p* = 0.12, *r* = 0.089).

The incidence of widening but not narrowing of the temporohyoid joint significantly increased with age (independent sample Mann–Whitney U test, *p* < 0.001/*r* = 0.29 and *p* = 0.09/*r* = 0.10, respectively). Both factors had a significant association with maximum temporohyoid grade (independent sample Mann–Whitney U test, *p* < 0.001/*r* = 0.54 and *p* < 0.001/*r* = 0.50), with increased prevalence of both widening and narrowing with increased grade. Widening was seen in 96.2% of horses with temporohyoid Grade 3, 76.9% of Grade 2, 10.8% of Grade 1, and 3.2% of Grade 0.

Four horses had fusion of the temporohyoid joint, all of which were unilateral, and three out of four had concurrent ipsilateral fractures of either the temporal (two horses) or styloid bone (one horse).

### 3.3. Remaining Hyoid Findings and Signalment

An ossified epihyoid was seen separate from the stylohyoid unilaterally in 41 horses and separate bilaterally in 71 horses (Table 1). Median age of horses with a separate epihyoid (9 years old) did not significantly vary (independent sample Mann–Whitney U test, *p* = 0.64, *r* = −0.28) from the age of horses with bilaterally fused/non-formed epihyoid (9 years old).

The basihyoid and thyrohyoid were fused in most horses and when separate were usually in young horses. All one-year-olds had separate thyrohyoid bones, and so did a minority of two-year-olds and a very small number of horses over two years of age. A significant difference (independent sample Mann–Whitney U test, *p* < 0.001/*r* = −0.31) in age was found between horses with a separate thyrohyoid present (median 2 years old, range 1–10) compared to the remaining population.

Separate centres of ossification of the lingual process were seen in predominantly young horses (median age 5 years old, range 2–11), with a significant variance in age from those without (independent sample Mann–Whitney U test, *p* < 0.001/*r* = −0.37).

Changes to the hyoid apparatus outside of the temporohyoid joint and those described above included arthropathy, deformation or fusion, and fracture. Twenty-five percent of the arthropathies were bilateral and predominantly positioned at the stylo-/epi-/cerato-hyoid joint; the remaining changes were predominantly unilateral or asymmetrical. Three of the four fractures were of the temporal bone, one was of the stylohyoid bone. Two horses had both fracture and deformation, two horses had both fracture and arthrosis, and six horses had both arthrosis and deformation. All fractures had ipsilateral Grade 3 changes of the temporohyoid joint, and three had ipsilateral fusion of the joint.

The horses with arthropathy (*p* = 0.02/*r* = 0.13), deformation (*p* = 0.007/*r* = 0.016), or fractures (*p* < 0.001/*r* = 0.21) of the remaining hyoid showed a significant increase in temporohyoid grade compared to those without.

### 3.4. Comparison of Hyoid Changes in Headshaking and Control Groups

No significant difference in maximum, right, or left temporohyoid grade was present between headshaking and control groups (independent sample Mann–Whitney U, *p* = 0.19/*r* = 0.077, *p* = 0.16/*r* = 0.082, and *p* = 0.24/*r* = 0.069, respectively). Group comparisons were also performed using multiple temporohyoid grade cut-off values, with no significant results (Table 1). The difference in temporohyoid grade between the right and left side was approximately similar between headshaking and control groups, with no significance found (Table 1 and Table 2).

Comparisons between widening, narrowing, and mineralisation of the temporohyoid joint also resulted in no significant association with headshaking (Table 2). A greater proportion of horses with widening of the joint were within the headshaking group compared to those without widening present; however, significance was not reached.

Comparisons were also performed for horses with fractures/luxations, deformation/fusion, or arthropathy of the remaining hyoid apparatus (Table 2).

Separate centres of ossification of the lingual process were seen at increased frequency in the headshaking group, and the remaining ossification centres were seen at increased frequency in the control group; however, none reached statistical significance.

### 3.5. Comparison of Headshaking Horses with No ‘Likely Relevant Finding’ and Control Group

Eighty-four of the headshaking horses had a ‘likely relevant finding’ present. The ‘likely relevant findings’ included dental (59), sinus (39), skull (16), middle ear (10), brain (5), or nuchal bursa (5) abnormalities.

The remaining 57 horses formed a subgroup of headshaking horses referred to as ‘no likely relevant findings’. The median age of the group was 8 years (range 3 to 15), with 26 mares and 31 geldings. Most horses were Dutch warmbloods or riding horses, accounting for 30 horses, and the remaining sample was mixed.

All comparisons of maximum temporohyoid grade, widening, and narrowing of the temporohyoid joint with this subgroup of horses had decreased *r*-values and odds ratios compared to those of all headshaking horses (Table 2). However, significance was not reached.

Assessment of fractures/luxations, deformation/fusion, or arthropathy of the remaining hyoid apparatus was limited in power, and no significant findings were identified.

A separate ossification centre of the epihyoid was seen at a significantly increased frequency in the control compared to test group; otherwise, no significant differences in the presence of ossification centres were seen (Table 2).

## 4. Discussion

Multiple findings of the hyoid apparatus at CT showed significant correlation with age, such as grade of temporohyoid osteopathy, mineralisation of the tympanohyoid cartilage, or changes (arthrosis, fracture, or deformation) in the remaining hyoid apparatus [11,12,22].

Multiple changes in the temporohyoid joint and particularly those associated with new bone formation of the styloid sheath (temporohyoid grade and widening) increased the odds ratio of headshaking. In most cases, measures of association and correlation were increased when horses with a ‘likely relevant finding’ were removed from the test group. However, statistical significance was not reached in any comparison. No correlation was noted between the degree of mineralisation of the tympanohyoid cartilage and the presence of headshaking. The distribution of additional changes in the hyoid was described, with no statistically significant association with headshaking.

The results suggest that changes in the temporohyoid joint, particularly new bone formation within the styloid sheath, may have importance in horses presenting with headshaking. However, a consistent or predictable relationship was not found, and a statistically significant relationship between the degree of new bone formation of the styloid sheath and headshaking was not reached so the results do not allow rejection of the null hypothesis.

Investigation of headshaking is challenging, as pathological lesions can be inconsistently associated with the clinical presentation. This is reflected in our examination of the temporohyoid joint where various findings appeared to show an association with headshaking but a direct statistically significant relationship was not found. In previous investigations of headshaking, 7.8% of horses included had temporohyoid osteopathy. If this is presumed to only include severe cases (i.e., Grade 3), the proportion is very similar to that found in our group of headshaking horses where 12 horses (8.5%) were affected [5]. In contrast a previous study of 33 horses with temporohyoid osteopathy, 4 showed signs of headshaking [16]. The reason for this inconsistent correlation of pathological lesions and clinical presentation is unknown. This case control study reviews findings in a large group of headshaking horses with respect to a control group to increase the likelihood of finding a significant association of characterisation of changes in the temporohyoid joints; however, a significant relationship was still not found. This may suggest a variable relationship to CT findings and clinical presentation and highlight the importance of complementary diagnostics to evaluate the relevance of temporohyoid joint changes.

**Figure 1:**
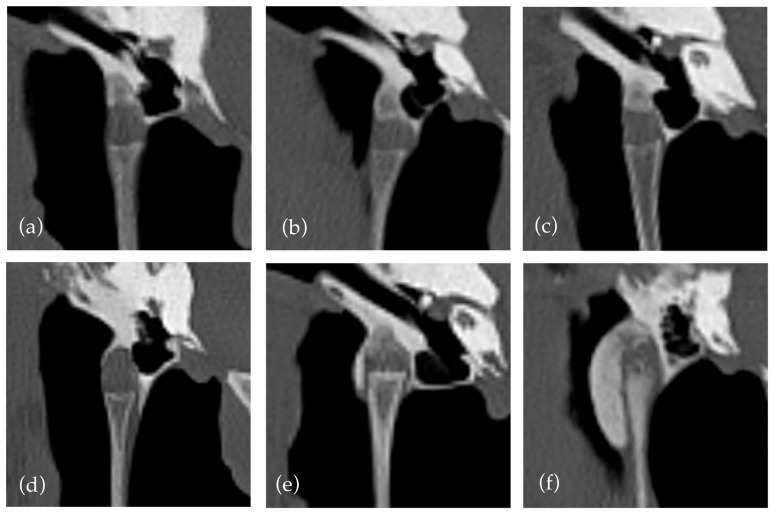
An illustration of the grades of temporohyoid joint. (**a**) Grade 0 shows no new bone formation. (**b**–**d**) all show Grade 1 temporohyoid joints; however, they illustrate the variation within this grade; (**b**) shows new bone formation not crossing the joint, while (**c**,**d**) show different degrees of new bone formation both crossing the medial aspect of the joint. (**e**) Grade 2. (**f**) Grade 3.

An existing grading system [17,21] was used to quantify new bone formation within the styloid sheath. The criteria were clarified that a grade two required new bone formation to cross the lateral and medial aspects of the joint. This decision was made due to a previous lack of clarity and the anatomy of the joint with a variably sloped medial aspect, which often affects the degree of new bone formation required to cross the joint. This is illustrated in Figure 1 where crossing new bone formation over the medial aspect of the joint can have limited relationship to the degree of new bone formation. The predisposition for medial distribution of new bone formation has been noted in previous papers [12]. The decision led to a relatively small number of horses with grade two changes (13) compared to grade one and three (157 and 26, respectively). This may suggest a limitation of the grading system to differentiate between mild and moderate cases, indicating a possible need to create a more even distribution between grades. Additionally, our interpretation classified horses as grade one if only medial new bone formation was present without extension around the joint, which may lead to underestimation/grading of some cases. The use of new bone formation crossing the joint is useful as it does not require measurement, but perhaps a grading based on the absolute rather than relative new bone formation may be more replicable and representative.

The utilised grading system [17,21] relies on the height of new bone formation within the styloid sheath. To offer a more comprehensive review of changes of the joints, we also examined narrowing, widening, and mineralisation of the temporohyoid joint.

Widening and narrowing of the temporohyoid joint showed strong correlation with temporohyoid grade and age, as well as insignificant correlation with the presence or absence of headshaking. Assessment of these features, not included in grading, added detail to the assessment of the temporohyoid joints beyond the height of new bone formation within the styloid sheath. Both widening and narrowing of the temporohyoid joint showed significant correlation with the temporohyoid grade and increased the odds ratio of headshaking, suggesting they may be an important aspect of temporohyoid osteopathy not assessed with the utilised grading system [17,21].

**Figure 2:**
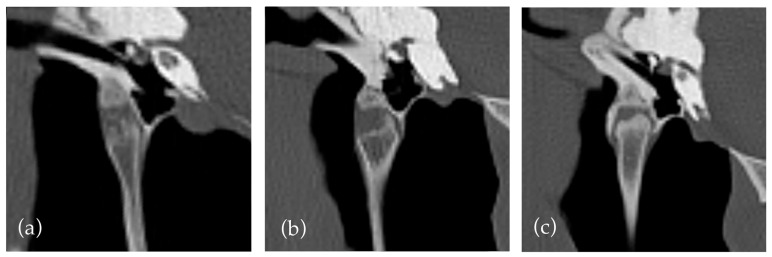
Illustrations of additional changes of the temporohyoid joint. (**a**) Mineralisation and narrowing. (**b**) Widened joint and mineralisation. (**c**) Widening, narrowing, and mineralisation.

However, the assessment of both features was subjective and challenging due to the influence of multiple factors, not accounted for in the study design. For example, subjective widening of the temporohyoid joint was observed in many horses with proliferative mineralisation of the synovial sheath and in horses with a widened or ‘club shaped’ joint in absence of other factors (Figure 2). Similarly, a narrowing or decrease in height of the temporohyoid joint can be due to a regular reduced height of the tympanohyoid cartilage or irregular ossification of the cartilage, so classification could be challenging.

Objective measures or criteria for the assessment of these changes in the temporohyoid joint are recommended for further analysis of the region. This has been reported previously with absolute values, and no adjustment for patient size was used [12]. Establishment of accurate, representative, and relevant measurements may be challenging; however, our findings indicate the possible importance of pursuing this route of further research.

Mineralisation of the tympanohyoid cartilage was observed in 46.6% of horses in the current study compared to 38.4% in earlier studies [11]. It showed no correlation with the headshaking. This suggests a relative lack of importance in the finding. However, the origin and distribution of mineralisation varied with some incidences of irregular bony contours of the adjacent bone (as mentioned above) and some discontinuous/central mineralisations within the cartilage. Without histopathology, it is challenging to differentiate the aetiology of the mineralisation. The broad criterion may also explain the slightly increased rate of detection in the current study.

Most changes in the temporohyoid joint showed significant association with age, in agreement with previous publications [11,22]. A general increase is expected with age so the maximum rather than mean grade between the right and left side was used, and left and right sides were analysed in isolation to assess the most severe changes present. The difference in temporohyoid grade was also analysed to assess the influence of asymmetrical temporohyoid joint changes. However, no correlation was found. The increase in ossification of the region with age is not unexpected and consistent with suggested degenerative aetiology of the changes [22]. Gas foci within the joint was the only finding of the region not to correlate with age; the reason for this is unknown.

Ossification of the styloid sheath has previously been linked to fusion of the temporohyoid joint and fracture of the temporal bone [17]. Fracture of the temporal bone and in one case styloid bone was seen; however, not at the proportion previously reported. This may be due to a different study population with differences in clinical presentation, breed, and use of the horses. The previous study reported a high number of affected Quarter Horses, which were minimally represented in our study group with six in the control and two in the headshaking groups [17,19].

Otherwise, relatively few changes in the remaining hyoid apparatus were found with a few instances of deformation/fusion of hyoid joints and most commonly arthropathy of particularly the stylo-epi-ceratohyoid joint. However, when present, changes in the remaining hyoid showed significant association with increased temporohyoid joint grade, suggesting an association of pathologies. This could be due to limitations in movements in some locations of the hyoid due to deformation or arthrosis causing additional stress and secondary changes elsewhere.

The epihyoid was separate in 38.6% of horses, which was similar to that previously reported (33%) [11]. Previously, few horses under 3 years of age were reported to have a separate epihyoid. A peak in frequency was noted at 3–5 years of age followed by a decrease with increasing age. This was suggested to be consistent with lack of ossification of the epihyoid in young horses and complete fusion with the stylohyoid in older horses. Our results do not fully support this hypothesis; the reason for this is unknown. The majority of our study population was warmblood, and breed was not reported in the previous study so heterogeneity may influence results [11]. Alternatively, technical factors affecting spatial resolution could also affect results. Further prospective or histological examination may offer more information.

A separate ossified epihyoid was observed at a significantly increased proportion of horses in the control compared to headshaking without ‘likely relevant findings’ groups. Other variables were examined, with no significant correlation of the presence of epihyoid ossification to styloid sheath new bone formation, and the age distributions of the test and control groups were similar. In absence of an overt confounding variable, a statistical anomaly is considered most likely, as no reason for a protective effect is known.

Otherwise, examination of the thyrohyoid and basihyoid ossification (including the lingual process) was predominantly similar to previously reported [11]. Although a greater range of older horses with separate centres of ossification of both the basihyoid and lingual process were identified, this may be due to a larger patient group, so detection of an increased number of relative outliers is expected. The ossification centres of the thyrohyoid and the lingual process of the basihyoid showed no correlation with headshaking.

This additional description of the development and normal anatomical variation of the hyoid apparatus is important to differentiate expected findings from possibly clinically important pathology [27,28].

The study has additional limitations. We did not obtain histopathological information about the hyoid apparatus. However, the described changes in the hyoid at CT have been reported in previous studies with histopathology, albeit with sometimes varying conclusions [11,12,22].

Additional tests and follow-up were not considered regarding the assessment of findings and possible relevance; this decision was made due to the retrospective nature of the study, limiting consistent additional information. Also, due to the diverse presentation and incompletely understood aetiology of headshaking, a gold standard approach is challenging, with sometimes conflicting results of anaesthesia and signs following treatment [5], which makes robust study design and search for relevant findings without confounding factors very challenging. Future studies with prospective design and a standardised diagnostic routine may provide more comprehensive information [4].

A challenging aspect of the study design was obtaining a representative test group of horses with headshaking. CT has high sensitivity for the detection of many findings not seen on other modalities [29,30,31]. However, the relevance of findings is often unknown or variable [4,5,7]. With limited knowledge available about the relevance of various findings, it is challenging to set objective exclusion criteria for a test group. We used previous literature to set considered criteria for a ‘likely relevant finding’. However, this could be improved with future study into the relevance of individual findings. Also, all comparisons were repeated for all headshaking horses or a smaller group with all horses with ‘likely relevant findings’ excluded to attempt to assess all findings as completely as possible.

The utility of CT as a diagnostic tool in the investigation of headshaking has been discussed with varying results from different studies [4,5,7]. A key point of discussion is the extent to which findings could be relevant to the presentation. The importance of findings is often unknown, so specific and targeted investigations are essential to further clinical understanding and increase in accuracy of imaging criteria for further studies. This could lead to increased clinical understanding and have a cumulative effect by informing criteria of future research studies.

Grading and scoring systems are useful to make reporting of findings more consistent and objective between examinations. We used a reported grading system to assess the temporohyoid joints, which enabled objective differentiation between the height of new bone formation within the styloid sheath [17,21]. However, multiple factors such as the width of new bone formation within the styloid sheath or changes of the tympanohyoid cartilage were not considered. The results suggest these factors may also show an association with headshaking. With increased knowledge of the anatomy of the temporohyoid anatomy [12] and range of findings reported in the current study, further works into a grading system or development of an AI evaluation tool that incorporates further potentially clinically important changes in the hyoid joint could be useful to differentiate cases for further research or clinical assessment. However, establishing an objective grading system with relevant weighting to different variables would be very challenging and require a multifactorial analysis and extensive objective analysis and measurement, which is outside the scope of the current examination.

## 5. Conclusions

The presentation, distribution, and frequency of CT changes consistent with temporohyoid osteopathy were described in a large population of horses with a significant correlation or association with age. Evidence of pathology of the remaining hyoid was also described with significant association with temporohyoid grade. Separate centres of ossification of the epihyoid, lingual process, and thyrohyoid were found in multiple horses. Multiple hyoid changes increased the odds ratios of headshaking; however, no consistent significant correlation or association was reached. The findings offer more information about the distribution of hyoid changes in a large patient group; however, further prospective and objective research is required to examine the association between changes in the hyoid apparatus and headshaking.

## Figures and Tables

**Table 1:** Frequency of changes of the hyoid apparatus in the total group, headshaking group, headshaking with no ‘likely relevant findings’ group, and control group. The classification ‘present’ groups unilateral or bilateral presence of a finding.

Finding	Classification	Total		Headshaking: All		Headshaking: No Likely Relevant Findings		Control	
		Frequency	Percentage (*n* = 290)	Frequency	Percentage (*n* = 141)	Frequency	Percentage (*n* = 57)	Frequency	Percentage (*n* = 149)
Maximum Temporohyoid Grade	Grade 0	94	32.4%	41	29.1%	14	24.6%	53	35.6%
	Grade 1	157	54.1%	78	55.3%	31	54.4%	79	53.0%
	Grade 2	13	4.5%	10	7.1%	6	10.5%	3	2.0%
	Grade 3	26	9.0%	12	8.5%	6	10.5%	14	9.4%
Difference in temporohyoid grade	0 Grades	222	76.6%	107	75.9%	42	73.7%	115	77.2%
	1 Grade	60	20.7%	31	22.0%	15	26.3%	29	19.5%
	2 Grades	8	2.8%	3	2.1%	0	0.0%	5	3.4%
Mineralisation of the tympanohyoid cartilage	Present	135	46.6%	64	45.4%	24	42.1%	71	47.7%
Widening of the temporohyoid joint	Present	55	19.0%	33	23.4%	14	24.6%	22	14.8%
Narrowing of the temporohyoid joint	Present	41	14.1%	22	15.6%	13	22.8%	19	12.8%
Fusion of the temporohyoid joint	Present	4	1.4%	0	0.0%	0	0.0%	4	2.7%
Gas within the temporohyoid joint	Present	7	2.4%	4	2.8%	2	3.5%	3	2.01%
Arthropathy	Present	16	5.5%	7	5.0%	3	5.3%	9	6.0%
Deformation/fusion	Present	13	4.5%	8	5.7%	4	7.0%	5	3.4%
Fracture/luxation	Present	4	1.4%	0	0.0%	0	0.0%	4	2.7%
Separate Epihyoid	Present	112	38.6%	48	34.0%	12	21.1%	64	43.0%
Basihyoid and thyrohyoid	Present	14	4.8%	3	2.1%	1	1.8%	11	7.4%
Separate centre of ossification of the lingual process	Present	38	13.1%	22	15.6%	9	15.8%	16	10.7%

**Table 2:** Statistical comparisons of hyoid changes in headshaking (HS) and control (C) groups, and headshaking horses with no likely relevant findings (HS-LRF) and control (C) groups. Any findings referred to group unilateral and bilateral cases together. Significant findings are indicated in bold.

Comparison		Statistical Test	Headshaking (HS) and Control (C) Groups		Headshaking Horses with no Likely Relevant Findings (HS-NLRF) and Control (C) Groups	
			*r* Value (*r*), or Odds Ratio (OR) and (95% Confidence Ratios)	*p* Value	*r* Value (*r*) or Odds Ratio (OR) and (95% Confidence Ratios)	*p* Value
Temporohyoid grade	Maximum temporohyoid grade	Independent samples Mann–Whitney U test	*r* = 0.077	0.19	*r* = 0.13	0.062
	Maximum temporohyoid grade over 2	Two-sided Fisher’s exact test	OR = 0.89 (0.4–2.0)	0.84	OR = 1.13 (0.41–3.11)	0.80
	Maximum temporohyoid grade over 1	Two-sided Fisher’s exact test	OR = 1.43 (0.73–2.83)	0.31	OR = 2.07 (0.92–4.67)	0.12
	Maximum temporohyoid grade over 0	Two-sided Fisher’s exact test	OR = 1.35 (0.82–2.21)	0.26	OR = 1.69 (0.85–3.38)	0.14
	Difference in temporohyoid grade	Independent samples Mann–Whitney U test	*r* = 0.011	0.85	*r* = 0.027	0.70
Other changes of the temporohyoid joint	Mineralisation of the tympanohyoid cartilage	Two-sided Fisher’s exact test	OR = 0.91 (0.58–1.45)	0.73	OR = 0.80 (0.43–1.48)	0.53
	Widened temporohyoid joint	Two-sided Fisher’s exact test	OR = 1.76 (0.97–3.21)	0.072	OR = 1.88 (0.88–4.00)	0.11
	Narrowed temporohyoid joint	Two-sided Fisher’s exact test	OR = 1.27 (0.65–2.45)	0.51	OR = 2.02 (0.92–4.43)	0.087
	Fusion of temporohyoid joint	Two-sided Fisher’s exact test	NA	0.12	NA	0.58
	Gas within the temporohyoid joint	Two-sided Fisher’s exact test	1.42 (0.31–6.46)	0.72	1.77 (0.29–10.88)	0.62
Pathology of the hyoid apparatus excluding temporohyoid osteopathy	Arthropathy	Two-sided Fisher’s exact test	OR = 0.81 (0.29–2.24)	0.80	OR = 0.86 (0.23–3.31)	1.0
	Deformation/fusion	Two-sided Fisher’s exact test	OR = 1.73 (0.55–5.43)	0.40	OR = 2.17 (0.56–8.40)	0.27
	Fracture	Two-sided Fisher’s exact test	NA	0.12	NA	0.58
Centres of ossification of hyoid apparatus	Separate ossified epihyoid	Two-sided Fisher’s exact test	OR = 0.69 (0.43–1.10))	0.15	OR = 0.35 (0.17–0.72)	**0.004**
	Separate thyrohyoid	Two-sided Fisher’s exact test	OR = 0.27 (0.07–1.0)	0.053	OR = 0.22 (0.03–1.78)	0.19
	Separate ossification centre of lingual process	Two-sided Fisher’s exact test	OR = 1.54 (0.77–3.06)	0.23	OR = 1.56 (0.65–3.76)	0.34

## Data Availability

Data availability is limited due to the clinical nature.

## Abbreviations

The following abbreviations are used in this manuscript:

CT	Computed Tomography
